# Flat variable liquid crystal diffractive spiral axicon enabling perfect vortex beams generation

**DOI:** 10.1038/s41598-023-29164-0

**Published:** 2023-02-10

**Authors:** Javier Pereiro-García, Mario García-de-Blas, Morten Andreas Geday, Xabier Quintana, Manuel Caño-García

**Affiliations:** CEMDATIC, ETSI Telecomunicación, Universidad Politécnica de Madrid, Av. Complutense 30, 28040 Madrid, Spain

**Keywords:** Liquid crystals, Adaptive optics, Optoelectronic devices and components

## Abstract

A transparent variable diffractive spiral axicon (DSA) based on a single LC cell is presented. The manufactured DSA can be switched between 24 different configurations, 12 convergent and 12 divergent, where the output angle is varied as a function of the applied topological charge. The active area of the device is created using a direct laser writing technique in indium-tin oxide coated glass substrates. Liquid crystal is used to modulate the phase of the incoming beam generating the different DSA configurations. The DSA consists in 24 individually driven transparent spiral shaped electrodes, each introducing a specific phase retardation. In this article, the manufacture and characterization of the tunable DSA is presented and the performance of the DSA is experimentally demonstrated and compared to the corresponding simulations.

## Introduction

Tunable lenses without moving parts are able to tune the focal distance by spatially modulating the light path. Several techniques to obtain focus change are proposed in literature^1–4^ and these have a large range of applications including displays^5^, communications^6^, telescopes^7^, eyeglasses^8^ or microscopy^9^. In all these applications it is desirable to reduce size and weight while decreasing the complexity of traditional lenses with movable parts^10^. The wide range of applications of adaptative elements have created an increasing interest in designing and manufacturing tunable lenses.

A way of tuning a lens without changing its curvature is by using a nematic liquid crystal (LC). When the incoming wavefront passes through the LC device, its phase is shifted according to the orientation of the LC molecules as a result of applying an external electric field. These LC devices are used to manufacture flat phase only devices not affecting other beam characteristics^11^. Depending on the distribution of this electric field along the anisotropic material, the lens can be convergent (positive) or divergent (negative)^12^. A plethora of different LC lenses such as hole–and-ring electrodes^13^, diffractive Fresnel lenses^14^, and complex multi-electrodes devices^15^ have been presented. They are all characterized by limited diameter size, focusing tuning range and/or manufacturing complexity.

An axicon is a conic lens that generates an annular pattern from an incoming collimated beam of light. Axicons were first described in literature^16^ as an element capable of imaging a point source into a range of points on a line segment along the optical axis. The length of this segment is known as depth of focus (DOF).

The axicon has been widely studied due to its number of applications. As an axicon can generate annular rings it can be used to trap particles inside its image^17,18^. Other applications can be found such as gonioscopy^19^, micro-drilling^20^ or tomography^21^.

While traditional systems control the depth of field by the apodization of the a pupil^22^, others employ tunable reflexive axicons that are capable of changing the length of the DOF^23,24^ to obtain same results. Alternatively, the DOF may be made tunable by adding an extra convex lens before an axicon. Manually changing the distance between the light source and the convex lens, or the distance between the axicon and the lens, introduces a change in the input angle of the axicon, resulting in a variation of the output angle, i.e. a variation of the DOF^25^.

Diffractive axicons based on liquid crystals have been recently presented^26–28^, most are based on reflective Spatial Light Modulators (SLMs)^27,28^, while others are founded on specifically designed electrodes, but with limited tuning liberty to adapt a perfect phase profile^26^.

In this work, the first specifically developed transparent LC diffractive spiral axicon (DSA) is presented, capable of emulating the behavior of a variable diffractive axicon, only employing 24 electrodes with full phase profile control. The presented DSA output light beam will carry an orbital angular momentum (OAM) characterized by a spiral wavefront. The central axis of the beam, after the light passes through the device, will contain all the phases ranging from 0 to 2π. Thus, a singular point, where a destructive interference occurs will be formed in all transversal planes^29^. Therefore, the developed device acts intrinsically as avortex beam generator.

A vortex beam is given a number, called the topological charge, depending on how many twists the light does in one wavelength. This topological charge may be positive or negative, depending on the twist handedness. The higher the number of the twist, the faster the phase of the light is spinning about the axis.

The conventional way of generating an optical vortex is by using a spiral phase plate (SPP)^29^. Illuminating a combination of an SPP and an ideal axicon with a Gaussian beam forms a Bessel Gaussian (BG) beam carrying OAM with a singularity in the middle^30,31^. BG beams can also be obtained by direct illumination of a DSA.

Adding a lens in a Bessel Gaussian beam leads to the Fourier transformation of the field^30,32^. This optical transformation results in a bright ring pattern with a dark hole. By changing the topological charge of the Bessel Gaussian beam, the whole system acts as a perfect vortex beam (PVB) generator. PVBs are vortex beams characterized by carrying different OAMs without modifying their intensity ring pattern radius^33,34^. PVB are relevant in many applications, especially in optical communications^35,36^ as the system can propagate different OAM while keeping constant the intensity pattern easing the light coupling and detection.

To the authors knowledge, the presented device is the first purpose built tunable DSA, consisting of a single LC cell with a spiral-shaped pixelated active area. The diffractive axicon can be switched between twenty-four configurations, corresponding with twelve topological charges (positive and negative) giving rise an equivalent set of 24 different corresponding emulated apex-angles. The device is characterized by its high fill factor, leading to high transmittance, as presented in the supplementary data.

### Theory

**Figure 1:**
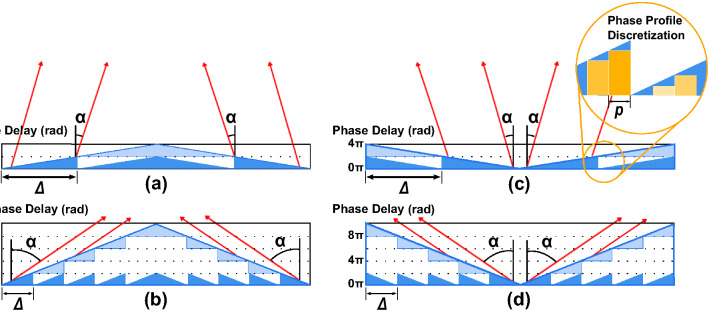
On the left, representation of two convergent blazed axicons wrapped over a 2π range. On the right, representations of divergent blazed axicons. The two different rows show two different grating pitches (Δ). The insert shows the discrete pixel distribution and pixel pitch (p).

An ideal refractive axicon has a conical structure. As in a Fresnel lens, a phase-wrapping over 2 can be performed^37^ resulting in a blazed diffractive axicon (Fig. 1a,c). By dividing the cone into periodic sections and applying a phase wrapping of 2π, a radial blazed phase profile is obtained. To increase the output angle (α) for a given wavelength (λ), the slope of the cone may be increased, corresponding to decreasing the blazed grating pitch (Δ) (Fig. 1b,d) similar to a linear diffractive blaze grating^38^.

1 $$\alpha ={tan}^{-1}\left(\frac{\uplambda }{\Delta }\right)\approx {sin}^{-1}(\frac{\uplambda }{\Delta })$$

A saw-tooth phase profile creates a 1st diffraction order beam with a diffraction angle given by the wavelength of the incoming beam divided by the width of the blazed pitch^39^. To make this phase structure addressable it has to be discretized into independentpixels. In this discrete structure, the minimum pitch, *i.e.* the maximum deviation angle, is obtained in a binary phase configuration consisting of electrodes with an alternating phase delay of 0 and π.

The previously described device produces a diffraction of first order conical waves, resulting in an annular shape in the far field^20^. Depending on the application, the zeroth order diffraction peak can be physically blocked. In the case of the implementation of the axicon with a SLM, it is usually eliminated by defocusing it at the processing plane with the computer hologram^40^.

When a light beam impinges an axicon its rays are bent at the same angle (*α*), relative to the lens normal, generating the conical wave. Depending on the blazed grating configuration the axicon can acts as a convergent or a divergent lens (Fig. 1a,c).

A convergent axicon generates a Bessel beam, sometimes so-called “non-diffractive” beam, with a transverse profile where the beam center does not change in amplitude and it is surrounded by concentric rings of lower intensity^41^. Bessel beams are characterized by an elongated depth of focus. Higher order Bessel beams carry OAMat the expense of losing energy in the central lobe^42^.

The *n*th-order electric field of the Bessel beam is defined by:

2 $$E\left( {r,\varphi ,z} \right) = AJ_{n} \left( {k_{r} r} \right)e^{{jk_{z} z}} e^{jn\varphi }$$

where *A* is the amplitude of the electric field and *J*_*n*_ is the *n*th-order Bessel function of the first kind; *k*_*z*_ and *k*_*r*_ are constants that represent the longitudinal and radial wavenumbers. *z*, *r* and $\mathrm{\varphi }$ correspond with the longitudinal, radial and azimuthal components, respectively. Thus, the formation of this electric field distribution is the result of the interference of plane waves whose wave vectors belong to a conical surface^43^.

**Figure 2:**
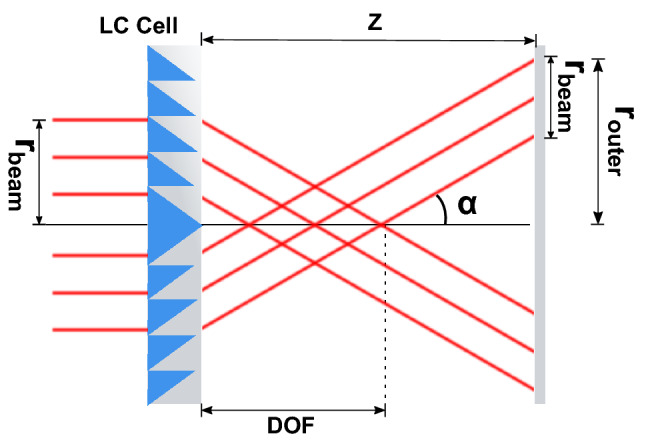
Blazed axicon ray diagram showing relevant parameters.

In a convergent configuration with a finite width (Fig. 2), the quasi-Bessel beam is formed leading to the resulting depth of focus (DOF), that will be a function of the beam radius (${r}_{beam}$) and the output angle (α):

3 $$DOF=\frac{{r}_{beam}}{tan\left(\alpha \right)}$$

The outer radius of the resulting annulus pattern (*r*) is given by:

4 $${r}_{outer}\left(z\right)=\left\{\begin{array}{c}{r}_{beam}-z\cdot tan\left(\alpha \right), z<DOF\\ z\cdot tan\left(\alpha \right), z>DOF\end{array}\right.$$

In a convergent configuration a disk pattern is generated before the end of the DOF, while the ring pattern is formed beyond this focal line. A divergent configuration, in which the incoming ray is deviated away from the propagation axis at angle ($\mathrm{\alpha }$), results in a ring pattern through all the propagation distance. The outer ring radius for a divergent configuration can be cast as:

5 $${r}_{outer}\left(z\right)={r}_{beam}+z\cdot tan\left(\alpha \right)$$

**Figure 3:**
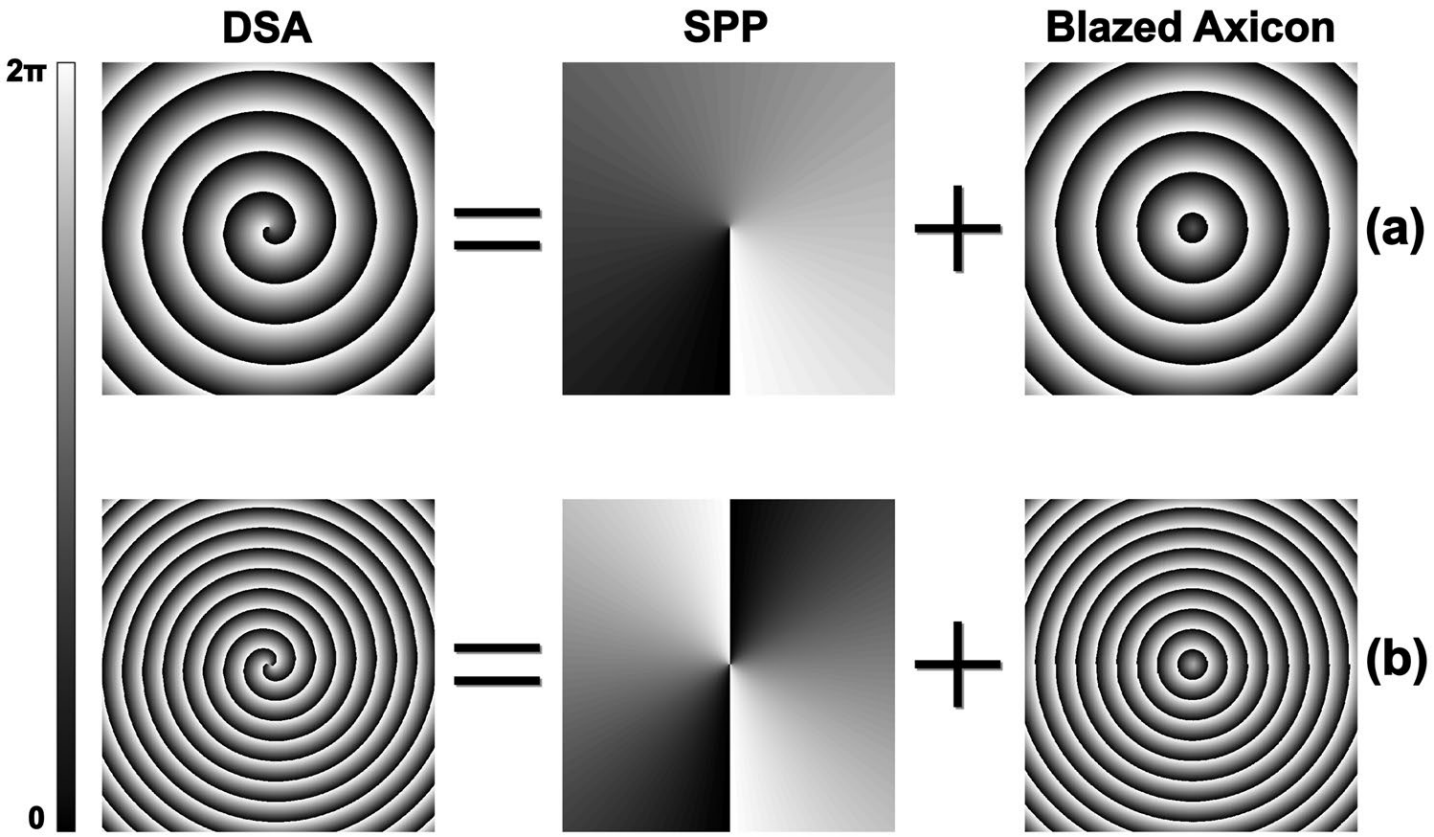
Scheme of the diffractive spiral axicon phase profile. It is obtained as the addition of a SPP and a blazed axicon for different topological charges. (**a**) l = 1. (**b**) l = 2. It is noticeable how a change in the DSA phase profile implies a simultaneous modification of the topological charge (l) and the axicon parameter (**a**).

The presented liquid crystal diffractive spiral axicon (DSA) is based on a diffractive structure made up of a spiral-shaped pixels (Fig. 3), easily connectable to an external electric field.

The spiral phase profile of the diffractive spiral axicon results from addition of the phase delay of one SPP with that of a diffractive axicon followed by a phase wrapping about 2π (Fig. 3). A similar approach has previously been used in the generation of polymer spiral phase mask axicons^33^.

The phase transmission function of a diffractive spiral axicon (DSA) is described as^33^:

6 $$t\left(r,\theta \right)=exp\left[\left(iar+il\theta \right)\right]$$

where *l* is the topological charge, *a* is the axicon parameter, *r* is the distance to the optical axis and $\uptheta$ is the azimuthal angle.

Theaxicon parameter and the output angle *α*, are determined by the blaze pitch ($\Delta$):

7 $$a=\frac{2\pi }{\lambda }\alpha =\frac{2\pi }{\lambda }{sin}^{-1}\left(\frac{\frac{2\uppi }{\Delta }}{\frac{2\pi }{\lambda }}\right)=\frac{2\pi }{\lambda }{sin}^{-1}\left(\frac{\lambda }{\Delta }\right)=k{sin}^{-1}\left(\frac{{k}_{r}}{k}\right)$$

where ${k}_{r}= \frac{2\uppi }{\Delta }$, is known as the radial wave number, and *k* is the light wave number.

The spiral pitch, $\Delta$, is equal to the blaze pitch. It is obtained by multiplying the number of spiral shaped pixels per period times the pixel pitch (*p)*. In other works^33^, where the axicon is not tunable, the spiral pitch, and thus *a*, is constant.

In the present tunable DSA, the number of pixels per period will depend on the device total number of pixels (*N*) and the selected topological charge (*l*), thus $\Delta$ is given by:

8 $${\Delta }_{l}=p\cdot \frac{N}{l}$$

We highlight that while *N* and *p* are design parameters, *l* is a reconfigurable parameter determined by the selected DSA addressing.

Thus, the axicon parameter and output angle of this tunable DSA (${a}_{l}$) can be expressed as:

9 $${a}_{l}=\frac{2\pi }{\lambda }{sin}^{-1}\left(\frac{\lambda \cdot l}{p\cdot N}\right)\iff {\alpha }_{l}={sin}^{-1}\left(\frac{\lambda \cdot l}{p\cdot N}\right)$$

The output angle of the DSA ${\alpha }_{l}$ will be dependent on the topological charge and number of electrodes. In a conventional refractive axicon, the output angle is determined by the apex angle and the material.

In the LC DSA the quality of the axicon will depend on the discrete approximation to the continuous axicon phase profile, while in a conventional the polishing quality is paramount^44^. Hence, in the developed flat device, the correct definition of the electrodes, the high number of electrodes and their correct calibration (ratio between applied voltage and phase shift produced) will be of great importance.

Combining Eqs. (6) and (9) and applying the paraxial approximation ${\alpha }_{l}={\mathrm{sin}}^{-1}\left(\frac{\lambda \cdot l}{p\cdot N}\right)\approx \frac{\lambda \cdot l}{p\cdot N}$, the transmission function of the developed tunable DSA can be expressed as:

10 $$t\left(r\right)\approx exp\left[il\left(\frac{2\pi }{p\cdot N}r+\theta \right)\right]$$

Thus, in the designed device the output angle (α_*l*_) and the topological charge (*l*) are inherently changing together.In the final device, as every pixel of the active area is independently driven, any phase profile can be shaped, resulting in a series of inter switchable diffractive axicons.

The presented DSA differs from conventional axicon as it intrinsically generates a vortex beam. Changing the phase profile configuration of the DSA modifies the topological charge of the vortex. Consequently in the DOF segment a high order Bessel Gauss beam, rather than a zeroth order Bessel beam, is formed^45^.

## Materials and methods

### Liquid crystal cells manufacturing

The LC diffractive spiral axicon (DSA) is made up of two indium tin oxide (ITO) substrates, in a sandwich-like configuration. One of the ITO substrates is used as a backplane and the other is pixelated using a Direct Laser Writing (DLW) technique. This ablation process is carried out by a UV laser mounted over a CNC controlled XYZ-stage (Lasing S.A). This system allows movements of the substrate in the XY plane while maintaining the ablation focus in the Z axis with a closed loop feedback^46^.

The active area consists of 24 continuous lines forming a set of concentric Archimedean spirals (i.e. pixels have the same width throughout the active area.), generating the contour of 24 pixels.

The pixel pitch (*p*), *i.e.* the designed width of the single spiral-shaped pixels, is 30 µm and the interpixel is < 3 µm^29^, leading to a device with maximum spiral pitch of ${\Delta }_{l}= 720\mathrm{ \mu m}$ (*l* = ± 1 and *N* = 24) and a fill factor better than 90%.

To assure a uniform alignment of the liquid crystal molecules and the switching plane, both ITO planes are covered with a polyimide PIA-2304 (Chisso Lixon, Japan) by spin coating (30 s @ 2500 rpm) and rubbed. The thickness of the LC cell is set to 7.2 µm using cylindrical silica spacers. Finally, the cell is filled with a high birefringence nematic liquid crystal, MDA-98-16002. The transmittance spectrum of the manufactured device is shown in the supplementary information.

The spiral structure of the pixels reaching the periphery of the active area eases the connection to the voltage driver. Interconnections between ITO pixels and the flex connector is done by using an Anisotropic Conductive Adhesive (Hitachi Chemical, Japan).

An inhouse designed 12-bit Pulse Width Modulation (PWM) driver is used for the individual pixel addressing^29^. A square 5Vpp, 51 Hz signal is modulated by programmable PWM signals, generating the desired RMS voltage for each pixel.

## Calibration and measurements

**Figure 4:**
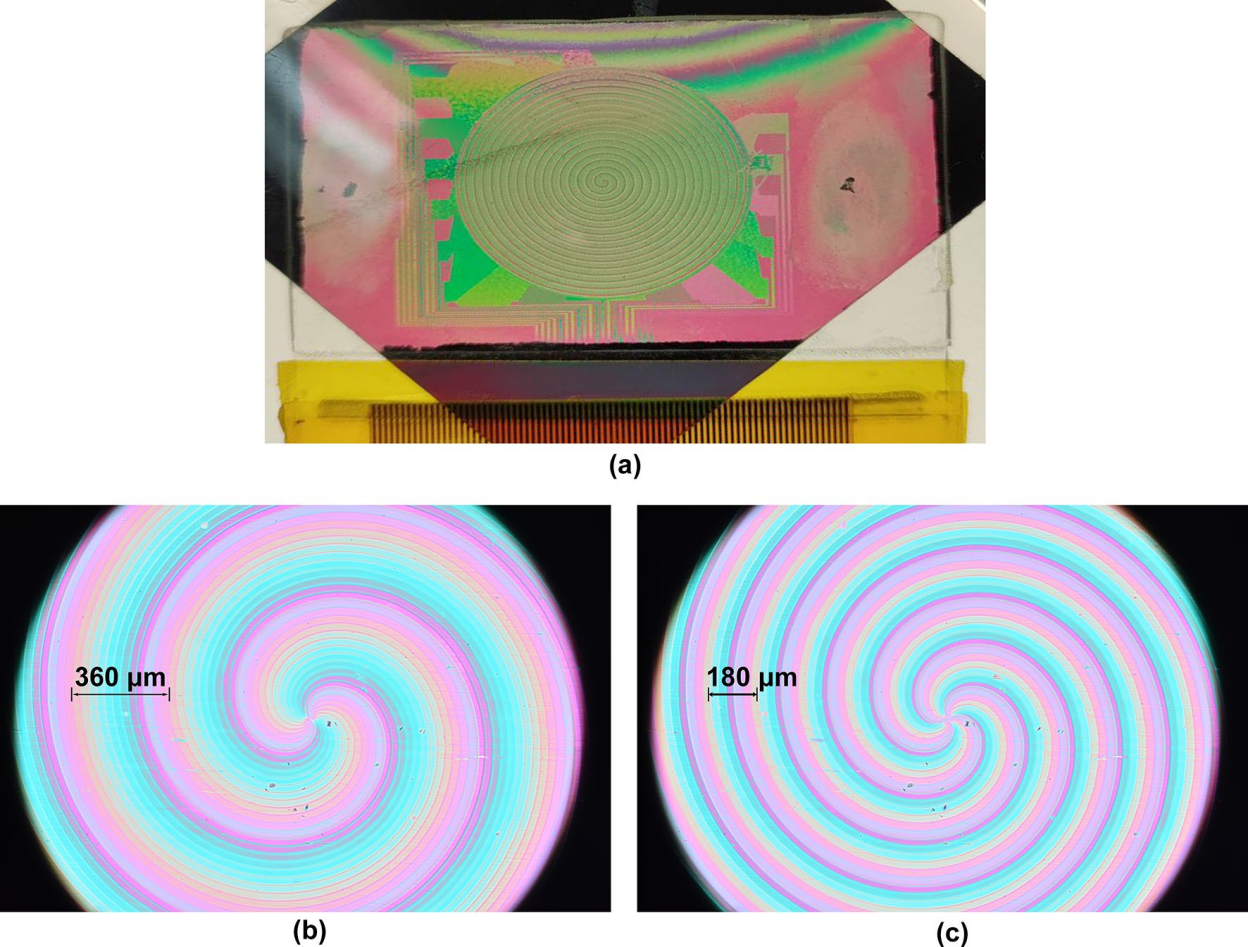
On top (**a**), the developed device. On the bottom, micrographs showing two different DSA configurations between crossed polarizers in white light illumination. (**b**) l = 2 ($\Delta =360\upmu m$). (**c**) l = 4 ($\Delta =180\mathrm{ \mu m}$).

A DSA picture is shown in Fig. 4a. The DSA was calibrated in a dark room at room temperaturebetween crossed polarizers at ± 45° with respect to the rubbing direction. The relationship between the applied RMS voltage signal and the phase delay induced by the LC, was determined using a He–Ne laser illumination (*λ* = 632.8 nm). The relation between the phase delay ($\delta$) and duty cycle (*dc*) has been approximated to:

11 $$\delta =A\cdot {e}^{-B\cdot dc}+C$$

as previously described^29^.

The developed DSA is a transmissive LC device. Thus, the phase profile introduced in the light polarized in the LC switching plane can be assessed by interference it with unaltered phase profile of the light polarizer perpendicularly hereto. Thereby, the phase profile becomes visible by placing the device between crossed polarizers, and observing the interference colors. In Fig. 4b,c two micrographs of the active area of the DSA between crossed polarizers are shown. In addition, the light beam TC is conserved during any radially symmetric phase transformation, such as light propagation^47^.

## Results and discussions

**Figure 5:**
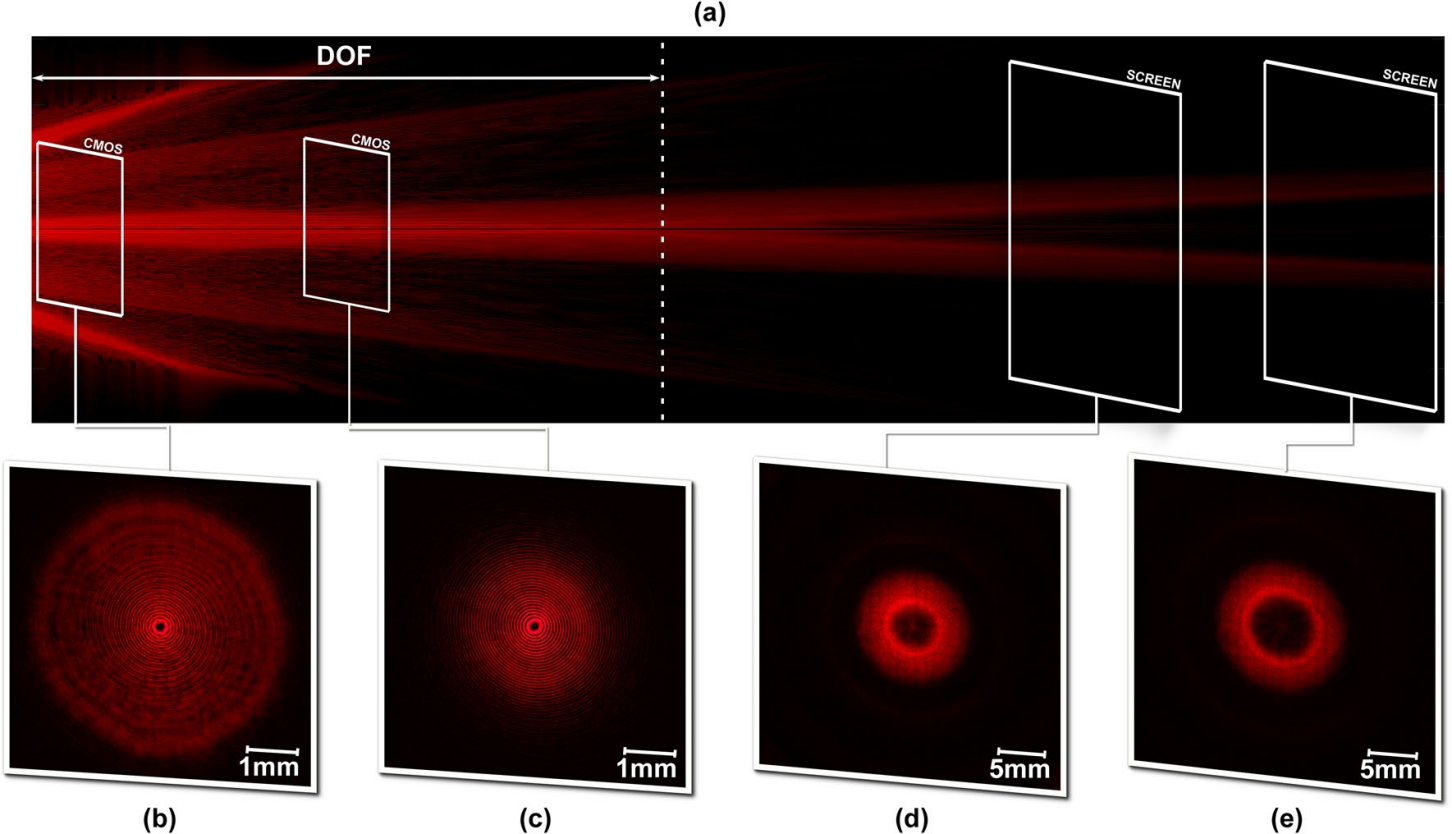
Beam evolution for a convergent DSA with l = 6. (**a**) Simulated beam propagation along 150 cm. (**b**–**e**) Intensity patterns experimentally measured at z = 25 cm, 50 cm, 125 cm and 150 cm, respectively. (**b**–**c**). The resultant pattern has been projected directly onto the CMOS sensor. (**d**–**e**). The resultant pattern has been projected onto an opaque millimeter screen. The intensity has been individually normalized in each measurement image.

In Fig. 5 the evolution of a Gaussian beam for a DSA with a fixed convergent configuration, with a topological charge of *l* = 6 corresponding to a spiral pitch of ${\Delta }_{l}=120\mathrm{ \mu m}$, has been simulated (as described in the supplementary data) and compared with measured results. Figure 5a shows the simulated cross-section of the propagation of the diffracted beam. One may clearly appreciate a zero-intensity segment along the optical axis within the DOF, corresponding to the singularity of the inherent generated vortex. Furthermore, this optical singularity is also manifested in the experimental Fig. 5b,c, where the output beam of the DSA is recorded directly into the camera sensor. In this area, the incoming beam is focused along the DOF area and the power distribution about the singularity is constant for a fixed *l*. The outcome is a diffraction pattern composed by positive and negative concentric interferences. The measured disk outer radius in Fig. 5b,c are 2.8 mm and 1.6 mm, in agreement with the theoretical values for an input beam radius of 4.2 mm.

Conversely, as the propagation distance is increased beyond the DOF area, the diffractive beam starts to diverge resulting in an annular ring-like shape (Fig. 5c,d). Consequently, the diameter of the ring increases with the propagation distance. The measured outer ring radius in Fig. 5d,e are 6.5 mm and 8 mm, in consonance with the theoretical values for an input beam with diameter 8.4 mm.

**Figure 6:**
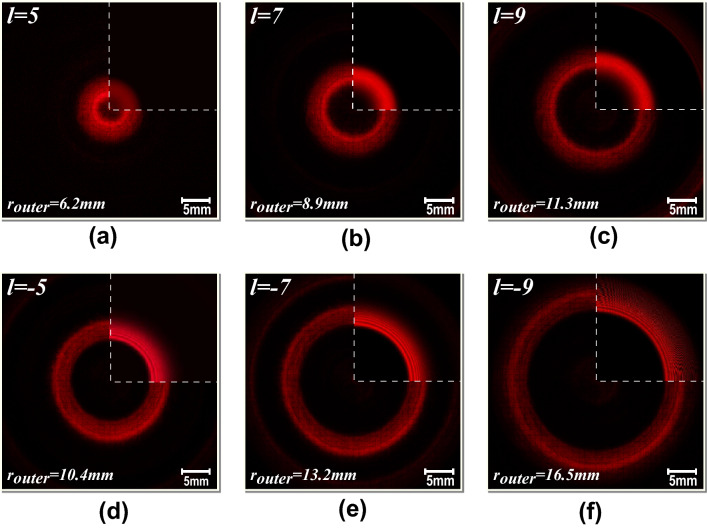
Experimental results of the DSA together with the corresponding simulations (top-right corner in the dashed lines). (**a**) Convergent configuration with l = 5. (**b**) Convergent configuration with l = 7. (**c**) Convergent configuration with l = 9. (**d**) Divergent configuration with l = − 5. (e) Divergent configuration with l = − 7. (**f**) Divergent configuration with l = − 9. The diffraction patterns are projected onto a millimeter and opaque screen at 150 cm from the DSA. The acquisition camera is focused onto the screen. The topological charge and the resulting outer ring radius are indicated in each image.

Figure 6 shows intensity patterns for different topological charges, at a fixed distance, illustrating the reconfigurability of the manufactured device.

In the first row of Fig. 6, positive topological charges are set. As these measurements reflect, there is a ring diameter growth as the topological charge is increased. The measured radii are consistent with the theoretical outer radius of Eq. (4), showing that the output angle increases with the DSA TC as predicted. The same conclusion can be drawn with respect to negative topological charges. Additionally, Fig. 6 also illustrates the concordance between the measurements and simulation.

In parallel, this figure confirms that the axicon high fill factor provides high efficiency in the diffraction process. Nevertheless, the efficiency decreases as the topological charge is increased.

**Figure 7:**
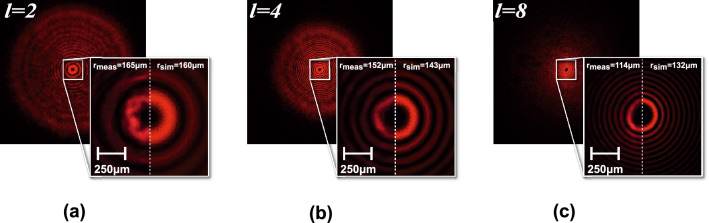
Bessel Gauss beam intensity pattern generated by the DSA at z = 50 cm for different topologies compared with the corresponding simulated results (superposed in the right part of zoomed section). (**a**) l = 2. (**b**) l = 4. (**c**) l = 8. The images show the light intensity projected directly onto the camera CMOS detector at 50 cm distance from the DSA. The radius of the for both simulations and measurements are overlaid.

In Fig. 7 the Bessel Gaussian beam generated by the DSA in the depth of focus area is presented. Accordingly, for different topological charges, there is a concentric ring pattern with a singular point of interference in the middle. In the zoomed sections the separation and width of the BG pattern rings changing with the topological charge can be observed. The simulated pattern for each topological charge is superposed in the right part of zoomed section of the Fig. 7, in line with the obtained results.

Perfect Vortex Beams (PVB) can be generated by implementing the Fourier transformation of a BG beam^30^ i.e. by inserting a converging lens after a DSA (in a convergent configuration) inside the DOF. Thus, the presented device may be applied in the generation of PVB. However, since the developed device simultaneously change the output angle and topological charge, it is required to change the focal distance of the converging lens for each PVB. This can be done either by replacing the lens or by using a reconfigurable flat multifocal lens such as the one we have presented previously^2^.

**Figure 8:**
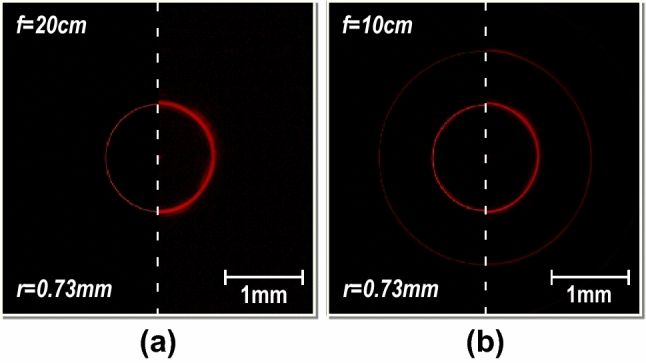
PVBs projected onto the camera CMOS sensor compared with the corresponding simulated results (superposed in the left part), generated by adding a lens to the DSA output at a distance z = 4 cm, with a focal distance f depending on the topological charge. (**a**) l = 4, f = 20 cm. (**b**) l = 8, f = 10 cm.

Figure 8 shows experimental measurements and simulations of such a convergent configuration, employing lenses with different focal distances depending on the topological charge, placed at a fixed distance within the DOF (z = 5 cm). One may appreciate that as the TC of the DSA is modified, a dark hollow with a bright constant radius is generated. Therefore, using the DSA as a tunable topological charge system and modifying the extra lens or using a multifocal lens, a perfect vortex beam could be generated.

## Conclusions

A variable diffractive spiral axicon device, based on a nematic liquid crystal structure, has been manufactured and demonstrated by experimental results. All these experiments are compared with their corresponding simulations. The manufactured DSA can be set to 24 different configurations, twelve convergent or twelve divergent, where the output angle and the topological charge are simultaneously tuned. A very high fill factor is obtained as the space between pixels (interpixel) is small compared to the pixel size since the active area is free of driving electronics. The optical singularity in the convergent configuration, focused along the optical axis, has been demonstrated. Simulations and experimental results show that the manufactured DSA in is able to perform perfect vortex beams in such a way, that the topological charge can be tuned without modifying the annular intensity size.

## Supplementary Information

Supplementary Information 1.

Supplementary Information 2.

Supplementary Information 3.

## Data Availability

All data generated or analyzed during this study are included in this published article (and its Supplementary Information files). The simulations codes are available upon request to the authors.

## References

[1] Li X (2016). Stretchable binary Fresnel lens for focus tuning. Sci. Rep..

[2] Geday MA, Caño-García M, Otón JM, Quintana X (2020). Adaptive spiral diffractive lenses—lenses with a twist. Ad. Opt. Mater..

[3] Ren H, Fan Y-H, Gauza S, Wu S-T (2004). Tunable-focus flat liquid crystal spherical lens. Appl. Phys. Lett..

[4] Algorri JF (2017). Tunable liquid crystal multifocal microlens array. Sci. Rep..

[5] Park S (2020). Electrically focus-tuneable ultrathin lens for high-resolution square subpixels. Light Sci. Appl..

[6] Zhang H (2018). Polarization-independent all-silicon dielectric metasurfaces in the terahertz regime. Photon. Res..

[7] Zhang S (2016). High efficiency near diffraction-limited mid-infrared flat lenses based on metasurface reflectarrays. Opt. Express.

[8] Hasan N, Banerjee A, Kim H, Mastrangelo CH (2017). Tunable-focus lens for adaptive eyeglasses. Opt. Express OE.

[9] Wang Z (2020). Flat lenses based on 2D perovskite nanosheets. Adv. Mater..

[10] Ghilardi M, Boys H, Török P, Busfield JJC, Carpi F (2019). Smart lenses with electrically tuneable astigmatism. Sci. Rep..

[11] Otón JM, Otón E, Quintana X, Geday MA (2018). Liquid-crystal phase-only devices. J. Mol. Liq..

[12] Lin Y-H, Wang Y-J, Reshetnyak V (2017). Liquid crystal lenses with tunable focal length. Liquid Cryst. Rev..

[13] Chiu C-W, Lin Y-C, Chao PC-P, Fuh AY-G (2008). Achieving high focusing power for a largeaperture liquid crystal lens with novel hole-andring electrodes. Opt. Express.

[14] Li G (2006). Large-aperture switchable thin diffractive lens with interleaved electrode patterns. Appl. Phys. Lett..

[15] Beeckman J (2018). Multi-electrode tunable liquid crystal lenses with one lithography step. Opt. Lett..

[16] McLeod JH (1954). The axicon: A new type of optical element. J. Opt. Soc. Am..

[17] Shao B, Esener SC, Nascimento JM, Botvinick EL, Berns MW (2006). Dynamically adjustable annular laser trapping based on axicons. Appl. Opt..

[18] Cheong WC (2005). Fabrication of efficient microaxicon by direct electron-beam lithography for long nondiffracting distance of Bessel beams for optical manipulation. Appl. Phys. Lett..

[19] Perinchery S, Shinde A, Fu C (2016). High resolution iridocorneal angle imaging system by axicon lens assisted gonioscopy. Sci. Rep..

[20] Kuang Z, Perrie W, Edwardson SP, Fearon E, Dearden G (2014). Ultrafast laser parallel microdrilling using multiple annular beams generated by a spatial light modulator. J. Phys. D Appl. Phys..

[21] Ding Z, Ren H, Zhao Y, Nelson JS, Chen Z (2002). High-resolution optical coherence tomography over a large depth range with an axicon lens. Opt. Lett..

[22] Breen T, Basque-Giroux N, Fuchs U, Golub I (2020). Tuning the resolution and depth of field of a lens using an adjustable ring beam illumination. Appl. Opt..

[23] Zhai Z, Cheng Z, Lv Q, Wang X (2020). Tunable axicons generated by spatial light modulator with high-level phase computer-generated holograms. Appl. Sci..

[24] Shen C, Hong Q, Zhu Q, Zu C, Wei S (2019). Holographic projection based on programmable axilens. Opt. Laser Technol..

[25] Zhai Z, Ding S, Lv Q, Wang X, Zhong Y (2009). Extended depth of field through an axicon. J. Mod. Opt..

[26] Algorri JF (2020). Positive-negative tunable liquid crystal lenses based on a microstructured transmission line. Sci. Rep..

[27] Sánchez-López MM, Moreno I, Davis JA, Gutierrez BK, Cottrell DM (2020). Double-ring interference of binary diffractive axicons. OSA Contin..

[28] Khonina, S. N., Porfirev, A. P. & Ustinov, A. V. Diffractive axicon with tunable fill factor for focal ring splitting. In (eds. Hrabovský, M., Sheridan, J. T. & Fimia, A.) 102331P (2017). 10.1117/12.2265017.

[29] Caño-García M, Quintana X, Otón JM, Geday MA (2018). Dynamic multilevel spiral phase plate generator. Sci. Rep..

[30] Liu Y (2017). Generation of perfect vortex and vector beams based on Pancharatnam–Berry phase elements. Sci. Rep..

[31] Arlt J, Dholakia K (2000). Generation of high-order Bessel beams by use of an axicon. Opt. Commun..

[32] Vaity P, Rusch L (2015). Perfect vortex beam: Fourier transformation of a Bessel beam. Opt. Lett..

[33] Guo Z (2020). Generation of perfect vortex beams with polymer-based phase plate. IEEE Photon. Tech. Lett..

[34] Ostrovsky AS, Rickenstorff-Parrao C, Arrizón V (2013). Generation of the “perfect” optical vortex using a liquid-crystal spatial light modulator. Opt. Lett..

[35] Zhu F (2017). Free-space optical communication link using perfect vortex beams carrying orbital angular momentum (OAM). Opt. Commun..

[36] Shao W, Huang S, Liu X, Chen M (2018). Free-space optical communication with perfect optical vortex beams multiplexing. Opt. Commun..

[37] Gourley K, Golub I, Chebbi B (2011). Demonstration of a Fresnel axicon. Appl. Opt..

[38] de Blas MG (2022). High resolution 2D beam steerer made from cascaded 1D liquid crystal phase gratings. Sci. Rep..

[39] Kim Y (2020). Large-area liquid crystal beam deflector with wide steering angle. Appl. Opt..

[40] Kuang Z (2009). Diffractive multi-beam surface micro-processing using 10ps laser pulses. Appl. Surf. Sci..

[41] Algorri J, Urruchi V, García-Cámara B, Sánchez-Pena J (2014). Liquid crystal lensacons, logarithmic and linear axicons. Materials.

[42] Khonina SN, Kazanskiy NL, Karpeev SV, Butt MA (2020). Bessel beam: Significance and applications—a progressive review. Micromachines..

[43] Nguyen HD, Sedao X, Mauclair C, Bidron G, Faure N, Moreno E, Colombier JP, Stoian R (2020). Non-diffractive Bessel beams for ultrafast laser scanning platform and proof-of-concept side-wall polishing of additively manufactured parts. Micromachines..

[44] High quality quasi-Bessel beam generated by round-tip axicon. https://opg.optica.org/oe/fulltext.cfm?uri=oe-16-17-12688&id=170373. 10.1364/oe.16.012688 18711507

[45] Curtis JE, Grier DG (2003). Structure of optical vortices. Phys. Rev. Lett..

[46] García de Blas M, Geday MA, Otón JM, Quintana Arregui X (2021). Two-dimensional digital beam steering based on liquid crystal phase gratings. Appl. Sci..

[47] Knyazev B, Cherkassky V, Kameshkov O (2021). “Perfect” terahertz vortex beams formed using diffractive axicons and prospects for excitation of vortex surface plasmon polaritons. Appl. Sci..

