# Supplementary material for: Flat variable liquid crystal diffractive spiral axicon enabling perfect vortex beams generation
Source: Sci Rep. 2023 Feb 10;13:2385. doi: 10.1038/s41598-023-29164-0 (PMC9918518; doi:10.1038/s41598-023-29164-0)
Supplement: Supplementary file 2 — Supplementary Information 2. [file 41598_2023_29164_MOESM2_ESM.pdf]

Legends for the supplementary videos

Flat Tunable Liquid Crystal Diffractive Spiral Axicon enabling Perfect Vortex Beams generation.

Javier Pereiro García*(1), Mario García de Blas*, Morten Andreas Geday(1), Xabier Quintana Arregui, Manuel Caño-García

CEMDATIC, ETSI Telecomunicación, Universidad Politécnica de Madrid, Av. Complutense 30, 28040 Madrid, Spain.

*Both authors contributed equally to this work.

(1) Email: javier.pereiro.garcia@upm.es; morten.geday@upm.es

1. 27cm_projected in camera sensor.mp4

This video shows the resulting light pattern projected in the camera sensor, generated by the Diffractive Spiral Axicon when its topological charge is tuned from l=-12 to l=12 in integer steps.

Link: https://drive.upm.es/s/bR7CIU91Ayofsw4

1. 150cm_projected in a paper screen.mp4

This video shows the resulting light pattern projected in a paper screen, generated by the Diffractive Spiral Axicon when its topological charge is tuned from l=-12 to l=12 in integer steps.

Link: https://drive.upm.es/s/e8eqS39mNZZXWsy
