# Supplementary material for: Flat variable liquid crystal diffractive spiral axicon enabling perfect vortex beams generation
Source: Sci Rep. 2023 Feb 10;13:2385. doi: 10.1038/s41598-023-29164-0 (PMC9918518; doi:10.1038/s41598-023-29164-0)
Supplement: Supplementary file 3 — Supplementary Information 3. [file 41598_2023_29164_MOESM3_ESM.docx]

**Supplementary Information**

Flat Tunable Liquid Crystal Diffractive Spiral Axicon enabling Perfect Vortex Beams generation.

Javier Pereiro García*(1), Mario García de Blas*, Morten Andreas Geday(1), Xabier Quintana Arregui, Manuel Caño-García

CEMDATIC, ETSI Telecomunicación, Universidad Politécnica de Madrid, Av. Complutense 30, 28040 Madrid, Spain.

*Both authors contributed equally to this work.

(1) Email: [javier.pereiro.garcia@upm.es](mailto:javier.pereiro.garcia@upm.es); [morten.geday@upm.es](mailto:morten.geday@upm.es)

**Experimental Setup**

Figure S1 show the set up to measure the Diffractive Spiral Axicon (DSA). A He-Ne laser with a wavelength of 632.8 nm is used as input ray beam. This beam is modified by a spatial filter with a beam expander attached to the laser, obtaining a collimated beam with a higher diameter. To reduce the diameter of the spot to the desired one (8.4 mm) a diaphragm is used. Then, the incoming beam pass through a polarizer to ensure the proper axis of polarization (90º, making it coincident with the alignment direction) Finally, the light goes through the DSA impinging in the camera sensor (Nikon D500^1^) which is mounted in a stage with three micropositioners.

For some measurements the pattern generated by the DSA was bigger than the sensor, thus the measurement was obtained by projecting the light into a millimeter screen. In this case, the camera was mounted along with its objective directly focused into the paper screen.


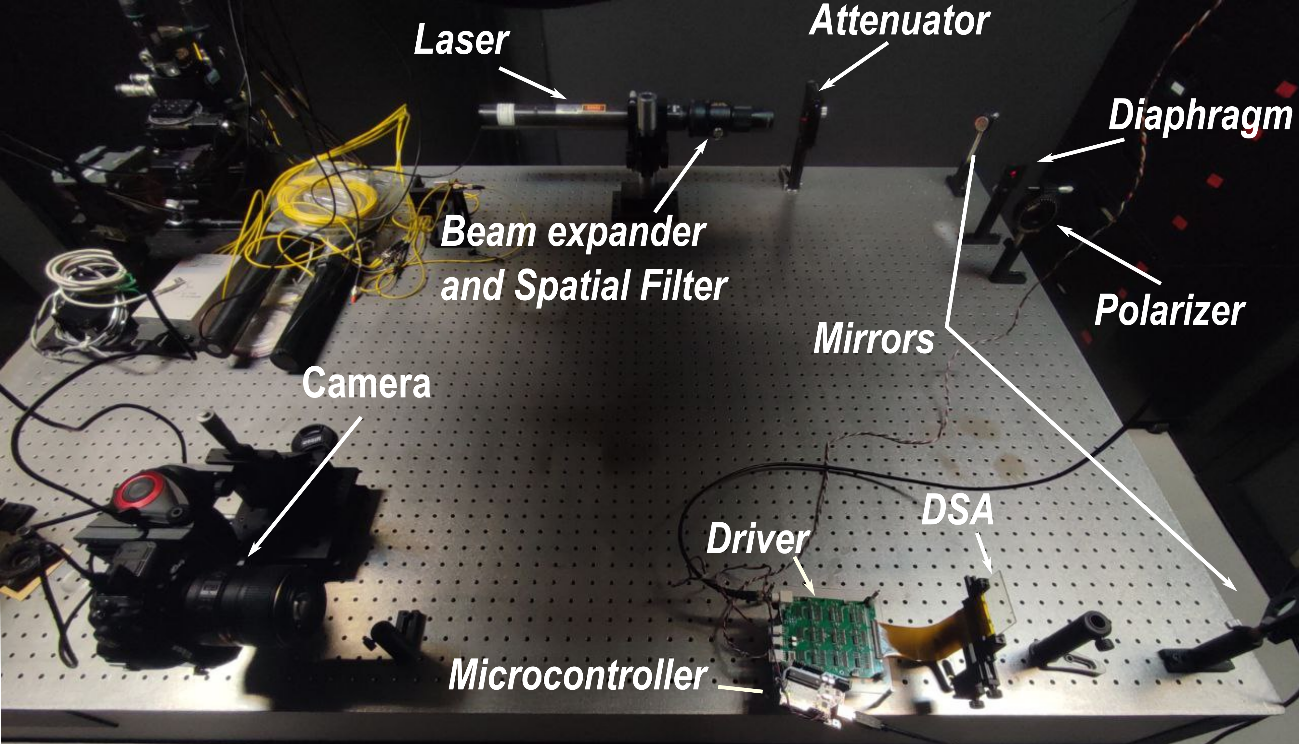


Figure S1 Experimental Setup.

The DSA configuration is tuned using an in house developed driver, that modifies the electric signals applied to the Liquid Crystal (LC) cell. This driver is controlled by using a Graphical User Interface (GUI) developed in LabVIEW ^2^. The communication between the driver and the PC is achieved by means of a microcontroller.

**Figures**

All figures have been created using Inkscape^3^ which is an open-source vector graphics editor. The measurements taken by the camera are obtained in .NEF format, then converted to .PNG format by using RawTherapee^4^ being later added imported to Inkscape.

**Simulations**

These simulations are founded on the Scalar Diffraction Theory, in particular the Angular Spectrum approach is used, for a detailed treatment of this theory the reader is referred to the work of Joseph W.Goodman ^5^.


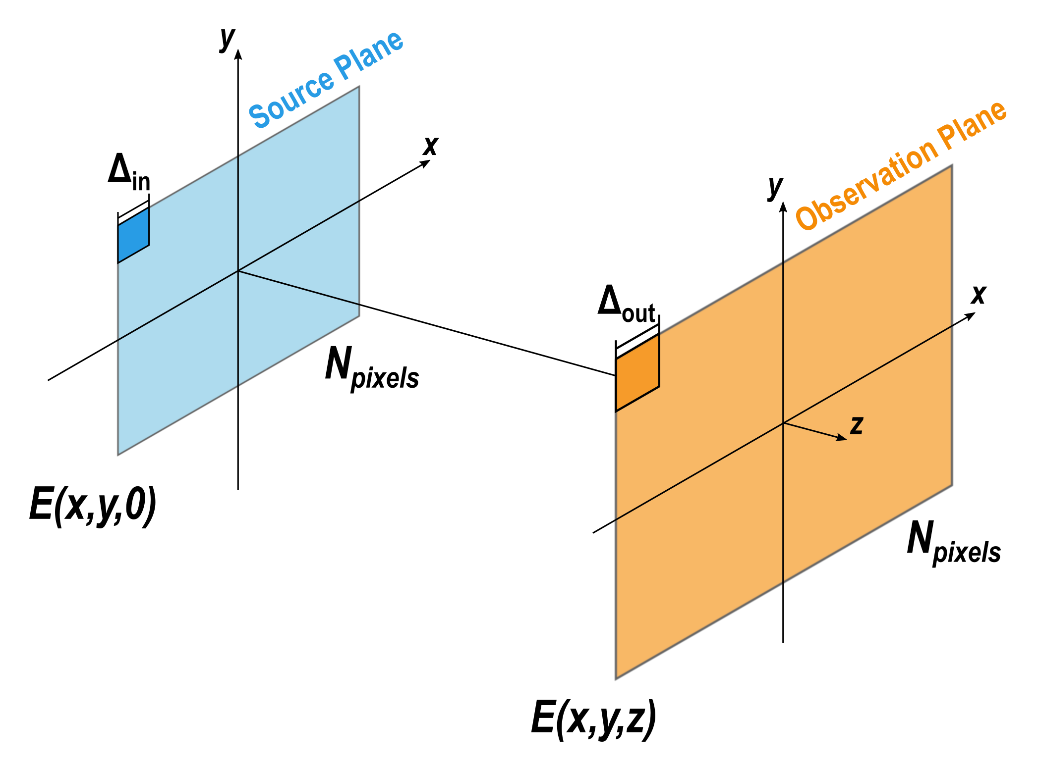


Figure S2 Angular Spectrum system scheme.

All simulations are carried in MATLAB R2018b^6^ under student license provided by the Technical University of Madrid (UPM). To implement the angular spectrum propagation methods, we used the functions provided by Jason D. Schmidt ^7^. By using this method, we can compute the resultant electric field at a specific distance by knowing the electric field at a source plane:

$$E(x_{obs},y_{obs},z)= e^{\frac{ik\left( m-1 \right)({x_{obs}}^{2}+{y_{obs}}^{2})}{2mz}}{Ftt2}^{-1}\left\{ e^{\frac{-i\pi^{2}2z({{(x}_{s}\frac{x_{s}}{N\Delta_{s}}N)}^{2}+{({{(y}_{s}\frac{x_{s}}{N\Delta_{s}}N)}^{2})}^{2})}{mk}}Ftt2\left\{ e^{\frac{ik\left( 1-m \right)({x_{s}}^{2}+{y_{s}}^{2})}{2mz}}E(x_{s},y_{s},0) \right\} \right\}$$

Where $k=\frac{2\pi}{\lambda}$ is the wavevector, $m=\Delta_{obs}/\Delta_{s}$ is the scaling parameter between the observation plane and the source plane, z is the propagation distance and N is the number of pixels of your simulation. Knowing that the observation plane pixel size $\Delta_{obs}$ relation with the source plane pixel size is given by the following relation:

$$\Delta_{obs}=\frac{\lambda*z}{N*\Delta_{obs}}$$

Please find below the process used to obtain the simulation showing the evolution of the beam propagation and the Perfect Vortex Beam (PVB) generated by using the DSA and different lenses.

Beam Evolution Cross Section

To obtain the beam evolution cross section attached below, the next process was carried out:

1. Define Simulation parameters: number of pixels, source plane px size, wavelength.
2. Create the source matrix built using a DSA matrix and a Gaussian Beam Matrix.
3. Pad with zeros the source matrix depending on the propagation distance. Hence, we will obtain an observation plane with a pixel size equal for every distance.
4. Calculate the observation electric field matrix using the angular spectrum method.
5. Crop the central column of this output matrix.
6. Repeat steps from 3 to 5 for every propagation distance.
7. Join together all the columns cropped to build a cross section of the beam propagation.


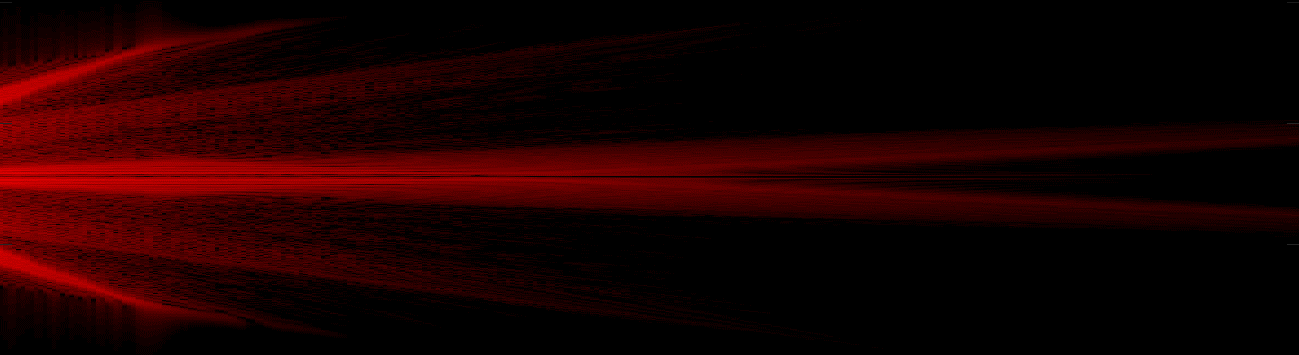


Figure S3 Simulation showing the beam propagation after a DSA with topological charge l=6.

PVB generation

To obtain the PVB please follow the next process:

1. Define Simulation parameters: number of pixels, source plane px size, wavelength.
2. Create the source matrix built using a DSA matrix with the desired topological charge (l=2, l=4, l=8) and a Gaussian Beam Matrix.
3. Calculate the Electric field at z=15cm. (This distance laid inside the Depth of Focus (DOF) for every topological charge) using the angular spectrum method.
4. Use this Electric field matrix as an input for a convergent lens with a focal distance dependent on the selected topological charge.
5. Calculate the output electric field at the focal point of the lens.


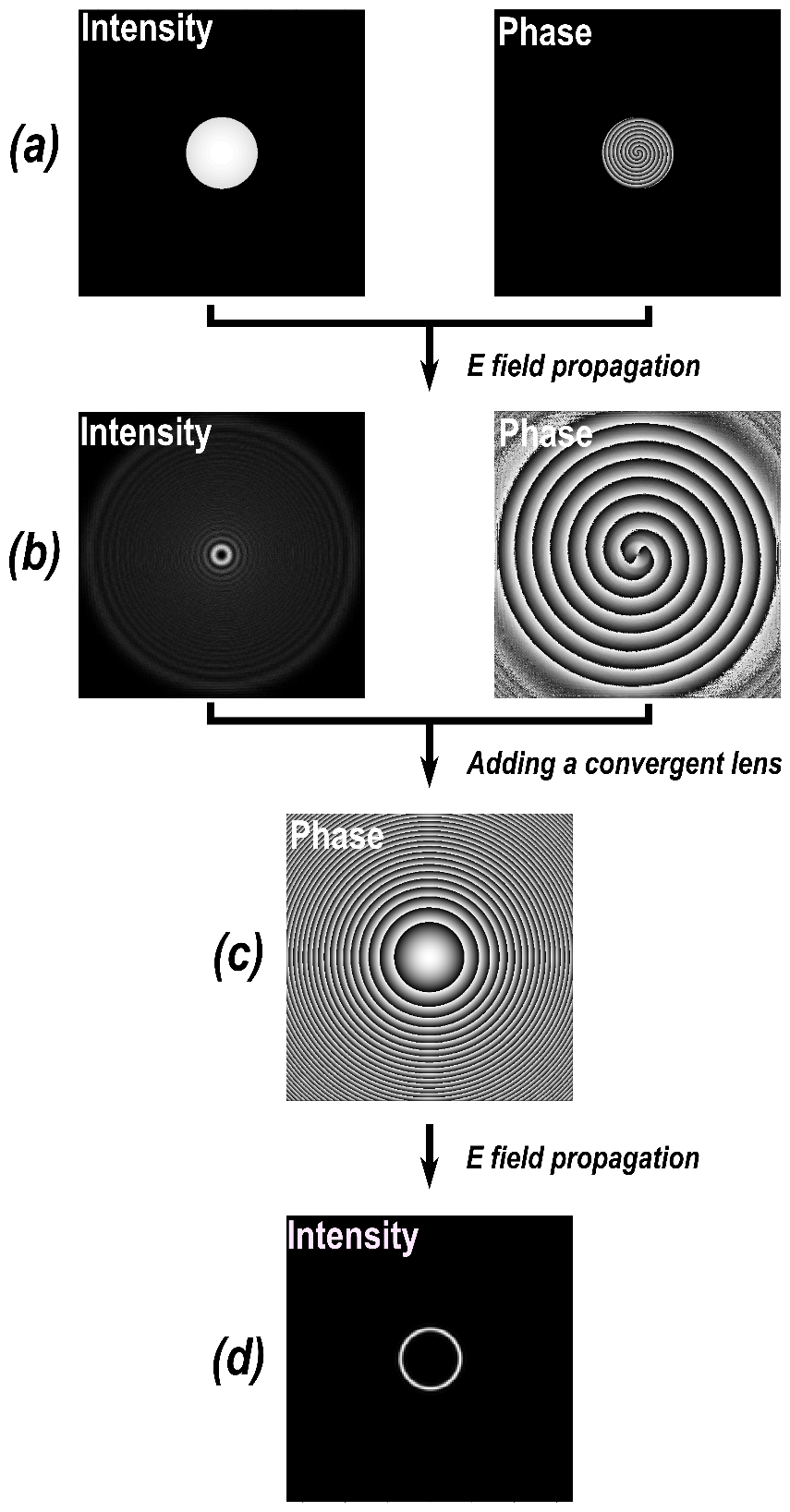


Figure S4 PVB Simulation process scheme: (a) Generate the DSA and the Gaussian Beam. (b) Calculate the electric field at z=15cm. (c) Lens Phase matrix added to the calculated electric field. (d) PVB intensity pattern calculated at the focal point of the lens.

**Device Transmittance**

Please find below the device transmittance for a range of wavelengths, obtained using a PERKIN-ELMER Lambda 2 customized Spectrophotometer.


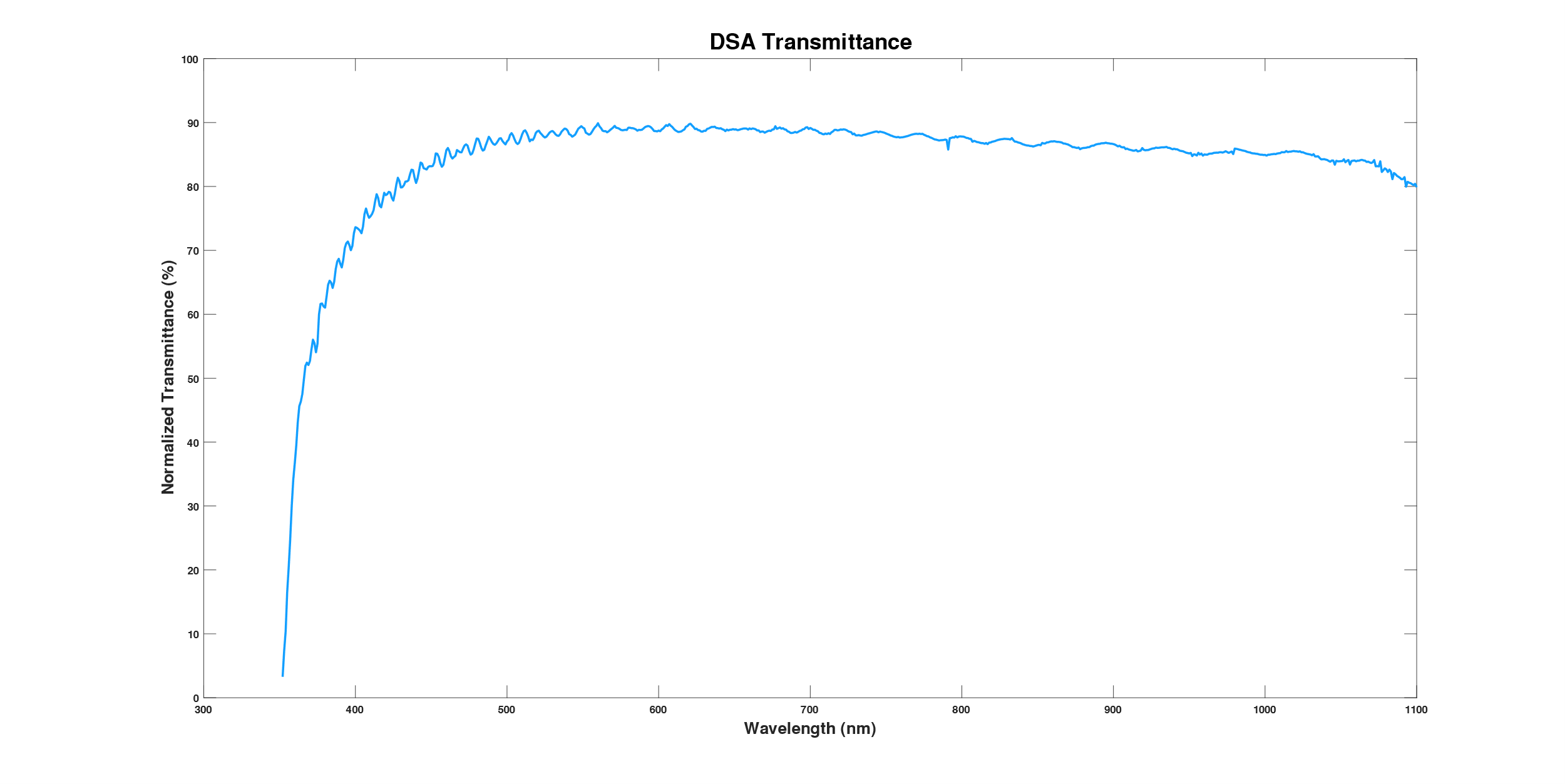


Figure S5 DSA transmittance data measured using the spectrophotometer.

The raw data obtained by the spectrophotometer is attached as supplementary file (“DSA_Spectrophotometer.xlsx”).

**References**

1. Nikon D500 | Cámara DSLR de formato DX | SOY RENDIMIENTO CONCENTRADO. https://www.nikon.es/es_ES/product/digital-cameras/slr/professional/d500.

2. What is LabVIEW? Graphical Programming for Test & Measurement. https://www.ni.com/en-us/shop/labview.html.

3. Developers, I. W. Draw Freely | Inkscape. https://inkscape.org/.

4. Home. https://www.rawtherapee.com/.

5. Introduction to Fourier Optics, 4th Edition | Macmillan Learning for Instructors. https://www.macmillanlearning.com/college/us/product/Introduction-to-Fourier-Optics/p/1319119166.

6. MathWorks - Creadores de MATLAB y Simulink - MATLAB y Simulink. https://es.mathworks.com/.

7. Numerical Simulation of Optical Wave Propagation with Examples in MATLAB | (2010) | Schmidt | Publications | Spie. https://spie.org/Publications/Book/866274?SSO=1.
